# Laboratory and epidemiology data of pertussis cases and close contacts: A 5-year case-based surveillance of pertussis in Indonesia, 2016–2020

**DOI:** 10.1371/journal.pone.0266033

**Published:** 2022-04-20

**Authors:** Sunarno Sunarno, Sundari Nur Sofiah, Novi Amalia, Yudi Hartoyo, Aulia Rizki, Nelly Puspandari, Ratih Dian Saraswati, Dwi Febriyana, Tati Febrianti, Ida Susanti, Khariri Khariri, Kambang Sariadji, Fauzul Muna, Yuni Rukminiati, Novi Sulistyaningrum, Dyah Armi Riana, Masri Sembiring Maha, Fitriana Fitriana, Vivi Voronika, Muamar Muslih, Mushtofa Kamal, Vivi Setiawaty

**Affiliations:** 1 Centre for Research and Development of Biomedical and Basic Health Technology, National Institute of Health Research and Development, Jakarta, Indonesia; 2 National Research and Innovation Agency (BRIN), Jakarta, Indonesia; 3 Centre for Research and Development of Health Resources and Services, National Institute of Health Research and Development, Jakarta, Indonesia; 4 Directorate General of Disease Prevention and Control, Ministry of Health, Jakarta, Indonesia; 5 World Health Organization Country Office for Indonesia, Jakarta, Indonesia; 6 Sulianti Saroso Hospital, Jakarta, Indonesia; Universidad Nacional de la Plata, ARGENTINA

## Abstract

Pertussis cases have been reported most frequently in developed countries, but they are predicted to be the most prevalent in developing countries. Indonesia, a developing country, routinely conducts case-based surveillance for pertussis. We reviewed the data on pertussis cases and close contacts based on clinical sample documents examined in the National Reference Laboratory for pertussis, Indonesia (2016–2020). Our objective was to analyze the laboratory and epidemiological aspects of pertussis cases and close contacts, particularly to evaluate the implementation of a 5-year case-based surveillance of pertussis in Indonesia. Data were collected from sample documents and annual laboratory reports between January 2016 and December 2020. We analyzed the proportion of pertussis cases and close contacts by geographic region, year, age, and sex. We used the χ^2^ test to correlate the laboratory and epidemiological data. In total, 274 clinical cases of pertussis and 491 close contacts were recorded in 15 provinces. The peak number of cases occurred in 2019, with a positivity rate (percentage of laboratory-confirmed cases) of 41.23% (47/114). Clinical cases were dominated by infants aged <1 year (55.5%), and 52.9% of them were aged <6 months. Similarly, 72.3% (68/94) of the laboratory-confirmed cases were infants. Both clinical cases and positivity rates tended to be higher in females (155 cases, 38.1%) than in males (119 cases, 29.4%). No confirmed cases were found in children aged ≥10 years, although positive results still occurred in close contact. Age-group and laboratory-confirmed cases were correlated (*p =* 0.00). Clinical and confirmed cases of pertussis occurred mostly in the early age group and may be lower in those aged ≥10 years, especially in confirmed cases. New policies are needed for pertussis prevention at an early age, as well as the application of serology tests to increase laboratory-confirmed cases in children aged ≥10 years.

## Introduction

Pertussis is a recurring health concern worldwide, in both developed and developing countries. The World Health Organization (WHO) has reported that most pertussis cases occur in developed countries. In 2019, China had the highest number of pertussis cases globally, with 30,027 cases; Japan, Russia, Australia, and India followed with 16,845, 14,407, 12,021, and 11,875 cases, respectively. China also recorded the highest number of cases in the previous year (2018) with 22,057 cases. After China, the number of cases was as follows: the USA (15,609), India (13,208), Germany (12,907), and Australia (12,555). In Indonesia, the WHO reported a relatively low number of pertussis cases, with only 27 cases in 2019 and 40 cases in 2018. However, in 2017, 2014, 2013, and several years ago, Indonesia had more than 1,000 cases [1].

Pertussis is a disease preventable by vaccination. The pertussis vaccine is commonly administered in combination with diphtheria toxoid and tetanus toxoid vaccines. Vaccine types and schedules vary among countries. Some countries have applied the whole-cell pertussis (wP) vaccine, a classic pertussis vaccine that has been used since the 1940s. Other (usually developed) countries have switched to acellular pertussis (aP) vaccines. The wP vaccine provides relatively longer protection against disease than the aP vaccine. In contrast, the aP vaccine causes milder side effects than the wP vaccine [2, 3]. Indonesia continues to use the wP vaccine, which is usually combined with diphtheria toxoid, tetanus toxoid, hepatitis B, and *Haemophilus influenzae* type b (pentavalent) vaccines. The pertussis vaccination program in Indonesia was scheduled to be implemented three times in children up to 6 months of age, followed by a booster shot at the age of 18 months [4]. Some countries have administered pertussis vaccines to pregnant women to prevent pertussis at an early age [5, 6]. However, pertussis is still persistent in some countries and has recently shown an increase in the number of cases, despite being vaccine-preventable [3, 7, 8].

Pertussis is caused by *Bordetella pertussis*. In addition to pertussis, *Bordetella parapertussis* has also been reported to cause diseases with clinical manifestations similar to those of pertussis. There are three phases of clinical pertussis: catarrhal, paroxysmal, and convalescent, with an incubation period of approximately 7–10 days (range, 5–21 days). The catarrhal stage lasts 1–2 weeks. At this stage, as signs and symptoms are not specific and resemble a common cold, pertussis diagnosis has not yet been established. Clinical diagnosis is usually established late in the paroxysmal stage, when whooping cough and post-tussive vomiting appear, while apnea is common in infants and neonates. The paroxysmal stage lasts for 4–6 weeks. The last stage is the convalescent stage, which lasts for 1–2 weeks, with clinical symptoms gradually recovering [3, 9, 10].

Laboratory confirmation can be achieved using various methods, including culture, polymerase chain reaction (PCR), serology, and oral fluid tests. The culture method is the gold standard for pertussis examination, although its sensitivity is limited. Culture is most sensitive if sample collection is conducted within 2 weeks of onset. The PCR assay is more sensitive and allows specimen collection up to 4 weeks after onset. However, PCR is less effective than serology when applied to adolescents and adults [11–13]. The Ministry of Health of the Republic of Indonesia routinely conducts case-based surveillance for pertussis. This activity involves the Public Health Emergency Operating Center (PHEOC), the Ministry of Health, health officers, hospitals, and laboratories, including clinicians, surveillance officers, and laboratory technicians. For this study, training was conducted for case finding, epidemiological investigation, sample collection, sample examination, and recording and reporting. We aimed to review the data of pertussis cases and close contacts based on the documents of samples examined in the National Reference Laboratory for pertussis in Indonesia. Our objective was to analyze the laboratory and epidemiological aspects of pertussis cases and close contacts, particularly to evaluate the implementation of a 5-year case-based surveillance of pertussis in Indonesia.

## Methods

### Data sourcing

Laboratory and epidemiological data regarding pertussis cases and close contacts were obtained from sample documents and annual laboratory reports as part of the case-based surveillance of pertussis in Indonesia from 2016 to 2020. All samples collected from patients with clinical diagnosis of pertussis and close contacts were delivered to Prof. Dr. Sri Oemijati Research Laboratory of Infectious Disease, Jakarta, Indonesia, as the National Reference Laboratory for pertussis. Regarding to the WHO guideline, a clinical or suspected case was defined as a person with a cough lasting ≥ 2 weeks, or of any duration in an infant or any person in an outbreak setting, without a more likely diagnosis and with at least one of the following symptoms: paroxysms (fits) of coughing, inspiratory whooping, post-tussive vomiting, or vomiting without other apparent cause, apnea (only in < 1 year of age) or clinician suspicion of pertussis [14]. Subsequently, the clinical samples were collected and delivered to the laboratory for confirmation. The clinical samples were a nasopharyngeal swab inoculated in Amies transport medium immediately after collection. The samples were delivered to the laboratory at 2–8°C and a sample document covering demographic and epidemiological data was attached. Unfortunately, not all sample documents were filled with complete data.

For epidemiological investigation, samples were also collected from both symptomatic and asymptomatic close contacts, even though the WHO guidelines did not recommend collecting samples from asymptomatic close contacts [14]. Regarding the guidelines, close contact was defined as a person who had face-to-face contact with a pertussis case, including household or family contact, direct contact with respiratory, oral, or nasal secretions from a confirmed case, and overnight stay in the same room with a patient. Unfortunately, no data were available to distinguish between symptomatic and asymptomatic close contacts in the present study.

In the laboratory, the samples were examined using the PCR method with three amplification targets (*IS481*, *ptxA-Pr*, and *IS1001*), following the guidelines of the European Centre for Disease Prevention and Control (ECDC) 2012 [15], with slight modifications. We extracted DNA using the QIAamp DNA Minikit (Qiagen) or Quick-DNA Kit (ZYMO RESEARCH). The fluorophore of PCR probes as well as PCR conditions following the ECDC guidelines were adjusted to the PCR machine (CFX 96; Bio-Rad), and polymerase enzymes were used. The ECDC guidelines can be used in several real-time formats [14]. Result interpretation refers to the algorithms described in the guidelines, with a cycle threshold (CT) value cut-off of 40. These PCR methods focus on *B*. *pertussis*, *B*. *parapertussis*, and *Bordetella* spp. identification. *B*. *pertussis* ATCC 12742 and *B*. *parapertussis* ATCC 15311 were used as the positive controls.

### Data analysis and ethical approval

We performed univariate analysis to describe the proportion of pertussis clinical cases and close contacts by geographic region, year, age, and sex using Microsoft Excel 2010. Bivariate analysis using the χ^2^ test was used to correlate laboratory and epidemiological data assisted by SPSS software, version 16.0. Statistical significance was set at *p* ≤0.05. The data analysis in this study was part of the routine national case-based surveillance of pertussis by the Ministry of Health. Individual data are fully anonymous, and the sample entity is replaced by the register number code of the sample. Therefore, ethical clearance was not required as stated by the Ethics Committee of the National Institute of Health Research and Development, Ministry of Health, Indonesia, in ethics statement No: LB.02.01/2/KE.557/2021.

## Results

### Case distribution

Based on sample examination in the National Reference Laboratory, 274 clinical cases of pertussis were suspected between 2016 and 2020 and were distributed in 15 out of 34 provinces in Indonesia (Table 1). Three provinces, East Java, South Sumatra, and West Java, have been continuously delivering clinical samples to the National Reference Laboratory. However, sample delivery in West Java began in 2017. Special Regions, Central Kalimantan and Yogyakarta, contributed the highest positivity rates (percentage of laboratory-confirmed cases) in 2019 (100%) and 2020 (67%), respectively. In 2019, a positivity rate of 50% was reported in samples from several provinces (South Sumatra, Bangka Belitung, and West Kalimantan). Statistical analysis by the χ^2^ test showed that there was no significant correlation between geographic region (Java island and non-Java island) and laboratory-confirmed cases (*p* = 0.07).

**Table 1:** Distribution of clinical and confirmed cases of pertussis in Indonesia during 2016–2020 by provinces.

No	Province	2016		2017		2018		2019		2020		Total	
		Cases	(%)*	Cases	(%)*	Cases	(%)*	Cases	(%)*	Cases	(%)*	Cases	(%)*
1	West Sumatera	3	0	-	-	-	-	-	-	-	-	3	0
2	Bengkulu	-	-	-	-	2	0	8	25.0	3	0	13	15.4
3	South Sumatera	7	0	12	41.7	3	33.3	22	50.0	9	33.3	53	41.5
4	Bangka Belitung	-	-	-	-	10	20.0	8	50.0	1	0	19	31.6
5	Jakarta	-	-	1	0	-	-	-	-	-	-	1	0
6	West Java	-	-	4	50.0	5	20.0	23	34.9	3	33.0	35	34.3
7	Central Java	-	-	-	-	-	-	6	16.7	-	-	6	16.7
8	Yogyakarta	-	-	-	-	-	-	15	40.0	3	66.7	18	44.4
9	East Java**	16	56.3	8	12.5	16	50.0	19	42.1	10	40.0	69	43.5
10	West Kalimantan	3	33.3	-	-	5	20.0	4	50.0	-	-	12	33.3
11	Central Kalimantan	-	-	-	-	3	0	4	100	-	-	7	57.1
12	South Kalimantan	7	42.9	23	13.0	1	0	2	50.0	-	-	34	20.6
13	East Kalimantan	-	-	-	-	-	-	1	0	-	-	1	0
14	West Sulawesi	-	-	-	-	-	-	-	-	1	0	1	0
15	Gorontalo	-	-	-	-	-	-	2	0	-	-	2	0

*% Laboratory Confirmed Cases

**including samples from regional laboratory

According to the data, the number of cases peaked in 2019, whereas the lowest number occurred in 2020 (Table 2). However, surveillance focused on the coronavirus disease 2019 (COVID-19) pandemic in 2020, which explains the decrease in pertussis cases. The positivity rate was 34.3% (94/274 clinical cases), with the highest rate of 41.2% in 2019. However, statistical analysis using the χ^2^ test revealed no significant correlation between the year of sample collection and laboratory-confirmed cases (*p =* 0.22). We found various positivity rates in close contact samples, ranging from 0% (2016 and 2020) to 29.9% (2017). In 2017, the data revealed that the carriers of pertussis were in large numbers, which could potentially transmit the disease to the surrounding vulnerable population. Unfortunately, no available data have shown the number or development of symptomatic close contacts.

**Table 2:** Proportion of clinical and confirmed cases of pertussis and close contacts by age, sex, and year of sample collection.

Variable	Clinical Cases			Close Contacts		
	n	Laboratory Confirmed	%	n	Laboratory Confirmed	%
**Age Category**						
<1 year	152	68	44.7	21	4	19.0
1–4 years	49	19	38.8	65	10	15.4
5–9 years	44	7	15.9	118	23	19.5
10–14 years	9	0	0	32	2	6.2
≥15 years	6	0	0	230	23	10
Unknown	14	0	0	25	0	0
**Sex**						
Male	119	35	29.4	210	28	13.3
Female	155	59	38.1	281	34	12.1
**Year**						
2016	36	13	36.1	34	0	0
2017	48	11	22.9	117	35	29.9
2018	45	13	28.9	42	1	2.4
2019	114	47	41.2	274	26	9.5
2020*	31	10	32.3	24	0	0
**Total**	**274**	**94**	**34.3**	**491**	**62**	**12.6**

*COVID-19 pandemic in 2020

### Case characteristics

According to the age groups, a distinct contrast occurred in which the infant category contributed to a higher proportion (Table 2). Of the 274 clinical cases, 152 (55.5%) were infants aged <1 year and 145 (52.9%) were aged <6 months. The highest percentage of positivity rate also appeared in the <1-year age group (44.7%), followed by 1–4-years and 5–9 years. In total, the proportion of laboratory-confirmed cases in children aged <1 year was 68 (72.3%) out of 94 total confirmed cases. No laboratory-confirmed cases were observed in children aged ≥10 years, whereas positive results were observed in close contact samples. Statistical analysis using the χ^2^ test showed a significant correlation between age (<10 years and ≥10 years) and laboratory-confirmed cases (*p* = 0.00).

Both clinical cases and positivity rates were higher in females (155 cases, 38.1%) than in males (119 cases, 29.4%). Of the 274 clinical cases, 155 (56.6%) were female, and 59 (62.8%) of the 94 laboratory-confirmed cases were female. However, statistical analysis using the χ^2^ test showed no significant correlation between sex and laboratory-confirmed cases (*p* = 0.14). In addition, there was no difference between females (12.1%) and males (13.3%) in the close contact samples.

### Further analysis

**Fig 1:**
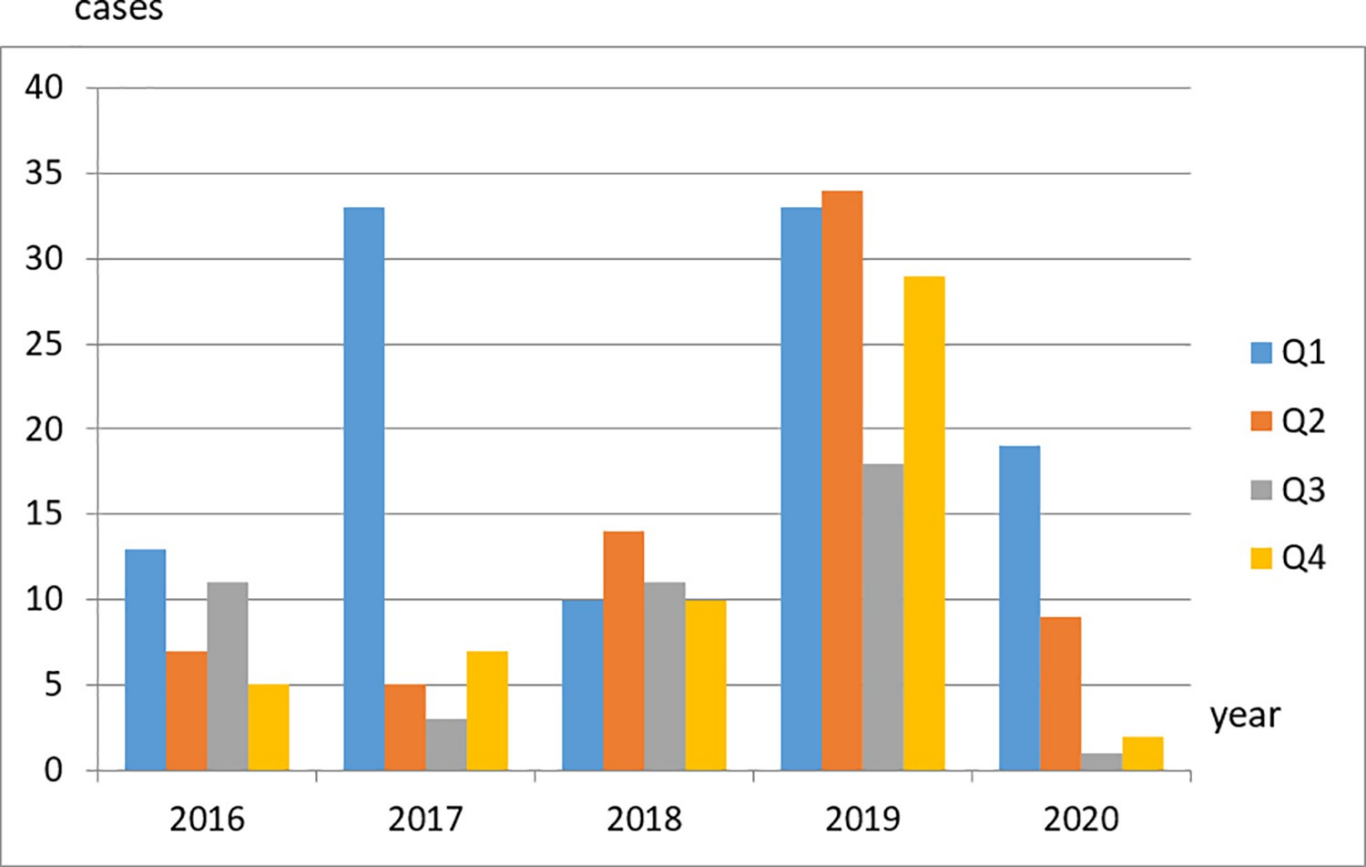
Proportion of pertussis cases in Indonesia between 2016 and 2020 based on period/quarterly (Q) event.

Further data analysis indicated that the number of cases tended to be higher in the first quarter of each year, although this did not apply in 2018 and 2019, when the second quarter had more cases than the first quarter (Fig 1). The proportion of cases in 2020 was high during the first quarter. However, among all cases (Q1–Q4), the lowest number of cases was identified in this particular year. These data support the possibility that the COVID-19 pandemic has had an impact on decreasing pertussis cases in 2020.

## Discussion

A total of 274 clinical cases of pertussis were identified in this 5-year national case-based surveillance in Indonesia from 2016 to 2020. These data do not represent real events because not all samples of clinical cases can be collected and examined in the National Reference Laboratory [16]. This is a limitation of the present study. However, these data provide evidence that pertussis persists in Indonesia, a developing country. The WHO predicts that pertussis cases are more prevalent in developing countries than in developed countries [17]. This prediction contradicts the WHO report, which showed that developed countries have the highest number of cases [1]. Table 1 shows the increasing trend of cases and yearly spreading area, with almost half of the provinces in Indonesia (15/34) providing samples for laboratory confirmation of pertussis. The data (Tables 1 and 2) exhibited a higher number of pertussis cases in 2018 and 2019 (45 and 114 cases, respectively) compared to the WHO data, which reported a lower number of cases in 2018 and 2019 (40 and 27 cases, respectively). In 2016 and 2017, the WHO reported that the number of cases was higher than the total number of examined samples [1]. This highlights the importance of coordinating and improving Indonesia’s surveillance system related to pertussis cases and laboratory examinations, especially recording and reporting.

The results of this study were similar to those of a previous study conducted by Peer et al., which showed a higher pertussis trend in females than in males in general [18]. Although, there was no statistically significant difference between female and male. The reason for this phenomenon remains unclear. It is assumed to be correlated with chromosome structure and hormonal factors in females. Most pertussis cases in many countries are also dominated by the infant age category [3, 18, 19], including Indonesia [16]. This high number of pertussis cases in infants was linked to individual susceptibility before obtaining a full dose of the pertussis vaccine and disease transmission from other household members [20, 21]. Several attempts have been made to reduce pertussis cases at an early age, including universal adult and adolescent vaccination; indirect protection of infants by vaccination of parents; and possibly others in close contact with the newborn, infant, and early infant vaccination, as well as maternal vaccination [3, 22]. Many countries have implemented maternal vaccination programs to reduce pertussis in early age infants [4, 23, 24]. However, the Indonesian Ministry of Health is yet to establish programs that involve previous attempts. Therefore, this review reinforces the recommendation to immediately apply preventive measures in cases of pertussis, particularly at an early age.

The positivity rate between children aged <10 and ≥10 years in this study was not proportional, as shown in Table 2. This was an evaluation of the laboratory confirmatory methods for pertussis in Indonesia. Currently, pertussis examination in Indonesia is based solely on PCR and occasionally on culture. In contrast, some studies have shown that culture and PCR are less sensitive than serology for pertussis case confirmation in adolescents and adults [25, 26]. Serology tests that are more suitable for children aged ≥10 years should be included in pertussis case confirmation in Indonesia as a new policy. Conversely, a high number of laboratory-confirmed close contacts indicates a high risk of disease transmission. Laboratory examinations of asymptomatic close contacts were not included in the WHO pertussis surveillance guidelines [14]. However, the data of close contacts (Table 2) can be used to predict disease transmission and to understand the indirect effect of age on examination results [21, 25]. This indicates the need for complete data to distinguish between symptomatic and asymptomatic close contacts, which were unavailable in this study. Other factors also contribute to this result, such as sample collection time (how long from onset), transport media and transport time (especially for culture), consumption of antibiotics, and mild symptoms [13, 14, 25]. These data were not available in this study, indicating the need to complete data to strengthen pertussis surveillance.

Pertussis does not occur continuously throughout the year. Fig 1 shows that most pertussis cases occur at the beginning of the year, that is, in the first and second quarters. This finding indicated that the occurrence of pertussis is influenced by seasonal and weather variations. England and the United States, for instance, reported a different pattern, whereby pertussis cases usually reached a peak in the third quarter [27, 28].

## Conclusion

Based on laboratory sample examination, we concluded that clinical and confirmed cases of pertussis occur mostly at early ages and may be lower in children aged ≥10 years, especially in confirmed cases. New policies are needed for pertussis prevention at an early age, as well as the application of serology tests to increase the number of laboratory-confirmed cases in children aged ≥10 years. This study also indicates the need to improve and strengthen national laboratory-based surveillance of pertussis. In addition, the Indonesian Ministry of Health is going to release a new surveillance guideline for pertussis that incorporates the findings and recommendations of this study.
